# Doppler ultrasound and contrast-enhanced ultrasound in detection of stent stenosis after iliac vein stenting

**DOI:** 10.1186/s12872-020-01840-3

**Published:** 2021-01-20

**Authors:** Hong Liu, Jing Wang, Yu Zhao, Zheng Chen, Dong Wang, Miao Wei, Fajin Lv, Xiaoping Ye

**Affiliations:** 1 Department of Vascular Surgery, the First Affiliated Hospital of Chongqing Medical University, No. 1 Youyi Road, Yuzhong District, Chongqing, 400016 China; 2 Department of Ultrasound, the First Affiliated Hospital of Chongqing Medical University, No. 1 Youyi Road, Yuzhong District, Chongqing, 400016 China; 3 Department of Radiology, the First Affiliated Hospital of Chongqing Medical University, No. 1 Youyi Road, Yuzhong District, Chongqing, 400016 China

**Keywords:** Contrast-enhanced ultrasound, Multidetector computed tomography venography, Iliac vein obstructive disease, Stent, Follow-up

## Abstract

**Background:**

To compare the diagnostic accuracy of Doppler ultrasound (DUS) with contrast-enhanced ultrasound (CEUS) for detection of iliac vein stent stenosis using multidetector computed tomography venography (MDCTV) as the reference method.

**Methods:**

Patients with iliac vein obstructive disease treated with nitinol stents (Smart Control, Cordis, USA) between January 2016 and December 2017 were consecutively included in this study. DUS, CEUS, and MDCTV were carried out in all patients within one week of each other at 1 year post stenting to investigate the presence of stent compression and in-stent restenosis (ISR).

**Results:**

The study included 139 patients (87 females; mean age 58 ± 15 years). For detecting stent compression, the kappa coefficient between the ultrasound modality of gray-scale imaging and MDCTV was 0.901, indicating very good agreement between these two modalities. ISR was detected in 50, 61, and 65 patients by DUS, CEUS, and MDCTV, respectively. DUS and CEUS (kappa = 0.449) and DUS and MDCTV (kappa = 0.516) had moderate agreement for ISR diagnosis, while for which CEUS and MDCTV (kappa 0.884) had very good agreement. The sensitivity and specificity of DUS and CEUS for diagnosing ISR were 63.1% and 90.8%, 87.8% and 97.3%, respectively.

**Conclusions:**

CEUS is probably superior to DUS in terms of diagnostic accuracy for the follow-up of patients with iliac vein stent stenosis.

## Background

Iliac vein obstruction may occur as a result of compression or following deep venous thrombosis [1]. Iliac vein stenting has been demonstrated to be safe and is associated with good clinical results [1–15]. Despite these therapeutic benefits, stent dysfunctions such as stent compression and in-stent restenosis (ISR) are common complications [5, 14, 16]. Significant compression of nitinol stents highly affects stent patency [17], and it has been reported that approximately 20 percent of stents will require reintervention for ISR [18].

Intravascular ultrasound (IVUS) remains the gold standard for iliac vein lesions [16, 18, 19]. However, IVUS is limited in routine follow-ups because of its high cost and invasive nature and thus is performed in only a minority of patients needed a secondary procedure. Noninvasive modalities, such as multidetector computed tomography venography (MDCTV), have replaced the traditional gold standard of invasive imaging and provide adequate information for both the anatomic results of stent placement and the changes in stented iliac veins [20].

Doppler ultrasound (DUS) has been widely used in the screening of venous diseases. However, its accuracy for the detection of iliac vein lesions is still controversial [21]. Contrast-enhanced ultrasound (CEUS) uses injected microbubbles as intravascular contrast and has been successfully used in the evaluation of endoleaks post EVAR [22] and TIPS follow-up [23]. To the best of our knowledge, the value of CEUS in the diagnosis of iliac vein stent stenosis has not been previously reported.

The purpose of this study was to compare the diagnostic accuracy of DUS with CEUS for detection of iliac vein stent stenosis using MDCTV as the reference method.

## Material and methods

### Study design and patients

The study received the institutional ethics committee approval, and all patients provided written informed consent. Patients who underwent endovascular treatment with nitinol stents (Smart Control, Cordis, USA) for iliac vein obstruction, either due to iliac vein compression syndrome (IVCS) or post-thrombotic syndrome (PTS), between January 2016 and December 2017 were eligible. The main inclusion criteria were IVCS patients associated with venous insufficiency (CEAP ≥ C3) or acute deep venous thrombosis (DVT), PTS patients with Villalta score > 4 and CEAP ≥ C3 and the age range was 18–80 years old. Exclusion criteria included a history of allergies to the ultrasound or MDCTV contrast agents used or unwillingness to provide written informed consent.

The routine follow-up surveillance program with DUS was performed at 1, 3, 6, and 12 months after the procedure and annually thereafter. CEUS and MDCTV were added to the surveillance at 12 months post stenting. DUS, CEUS and MDCTV imaging were performed within one week of each other.

### DUS and CEUS

The ultrasonographic examinations were performed by a single ultrasonologist who was a specialist in vascular ultrasound and was blinded to all other imaging results. All patients underwent intestinal preparation with overnight fasting to reduce bowel gas.

A Philips IU 22 with a 2–5 MHz curvilinear-array transducer (C5-1, Philips Medical Systems, USA) was used to perform the examination. Gray-scale imaging was applied to evaluate the existence of narrowing of the stent in the longitudinal and transverse views. The proper depth of penetration was optimized, and the overall gain and TGC were adjusted to clearly visualize the stents.

Color flow imaging was used to interrogate the luminal stenosis or occlusion. The color scale and color gain were adjusted to make sure the flow was shown in the lumen without saturating the stent walls and the surrounding tissues.

After the unenhanced imaging, CEUS was performed at a low mechanical index of 0.06. The tissue harmonic imaging mode was adopted to maximize the diagnostic effect of baseline ultrasound. Once the contrast agent (SonoVue; Bracco, Milan, Italy) was injected into the left dorsalis pedis vein as a single bolus of 1.2 ml followed by a 5 ml saline flush, the analysis archiving system were activated. The entire stent was scanned by axial and longitudinal for at least 4 min after the injection.

### MDCTV

MDCTV were performed using a 128-slice, dual-source CT system (SOMATOM Definition Flash, Siemens, Germany). The thickness of the reconstruction slice was set at 1 mm, and the spiral pitch was set at 0.85 mm. In total, 400 ml of saline-diluted contrast agent (37% iodine, iopromide, Ultravist, Bayer; the dilution ratio was 1:9) was intravenously administered in a bolus through bilateral dorsalis pedis veins at the same flow rate of 1–1.5 ml/s. Real-time bolus tracking was carried out at inferior vena cava (IVC) and used to synchronize the contrast passage with the venographic data acquisition. The trigger threshold was 100 HU, and scanning was delayed 5 s after triggering. The scan extended from the ankle to the diaphragm. The raw data were uploaded to a workstation (SIEMENS Healthcare, syngoMMWP VE40A) for image reconstruction.

### Image analysis

All the ultrasound images were reviewed by two different ultrasonologists, and two different radiologists reviewed the MDCTV examinations, all reviewers were blinded to the history of patients and other imaging results. The degree of stent compression was evaluated by calculating the percentage of diameter reduction of the patent lumen of the stent (diameter of the stent at the narrowest segment/diameter of the stent at the normal segment × 100%) [17]. The degree of stent compression was defined as “significant” if the luminal collapse was 50% or more and as “insignificant” if the luminal collapse was below 50%. ISR was defined as the presence of a focal or diffuse filling defect (neointimal hyperplasia or thrombosis) that separated the column of color Doppler blood flow or contrast material within the stent from the stent wall. Complete stent occlusion was diagnosed when no color flow signal on DUS or no microbubbles on CEUS or no contrast on MDCTV was observed.

### Statistical analysis

SPSS version 24.0 (IBM Corp, Armonk, NY) was used for statistical analyses. The agreement between different imagines for the detection of stent compression and ISR was evaluated with kappa statistics. Values less than 0.4, between 0.41 and 0.6, between 0.61 and 0.8 and above 0.81 indicated poor, moderate, good and very good agreement, respectively. The diagnostic accuracy was assessed by calculating the sensitivity and specificity.

## Results

### Patients

The study enrolled 139 consecutive patients (52 males, 87 females), with a mean age of 58 ± 15 years, who underwent endovascular treatment for iliac vein obstructions. Of these patients, 62 patients had nonthrombotic IVCS, 49 had IVCS with DVT, and 28 had PTS. The devices used were all nitinol stents (Smart Control, Cordis, USA) with a diameter of 12 to 14 mm, which were implanted in left iliac vein. All patients completed the protocol, and no adverse reactions to the ultrasound contrast agent or nonionic iodinated contrast agent were observed.

### Image analysis

**Fig. 1:**
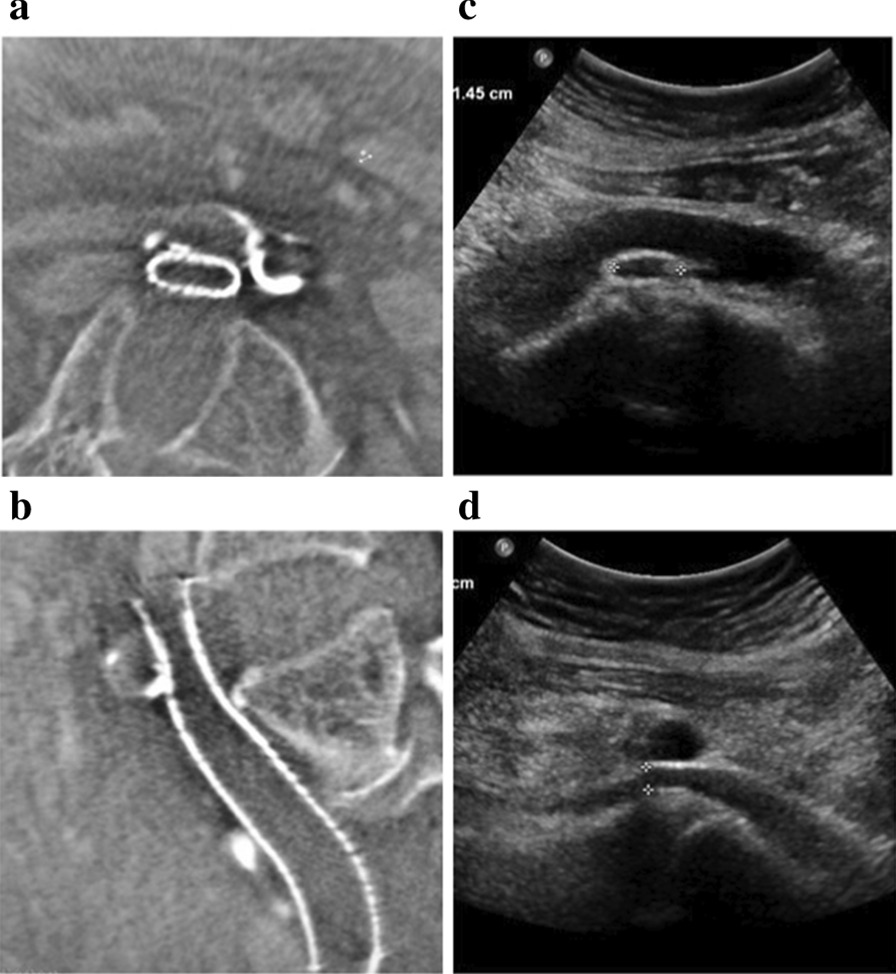
Follow-up MDCTV (**a**, **b**) and gray-scale imaging (**c**, **d**) showed significant stent compression in a 57-year-old female patient. In this patient, the upper end of the stent was compressed more than 50% by crossing iliac artery

Among the patients evaluated by MDCTV, 71.2% (99/139) had stent compression, including 22 patients with significant compression and 77 patients with insignificant compression. The most common site of stent compression was the proximal end of the stent (86.9%) caused by the right common iliac artery (Fig. 1a, b). MDCTV revealed stents with any degree of ISR in 46.8% (65/139) of the patients, including occlusion in 2.9% (4/139) of the patients.

Based on gray-scale imaging (Fig. 1c, d), significant stent compression was detected in 15.1% (21/139) of the patients, and insignificant stent compression was detected in 56.8% (79/139) of the patients. DUS and CEUS detected stents with any degree of ISR in 36.0% (50/139) and 43.9% (61/139) of the patients and occlusion in 4.3% (6/139) and 2.9% (4/139) of the patients, respectively.

### Agreement between different imaging modalities

The coincidence rate of gray-scale imaging and MDCTV in diagnosing the degree of stent compression was 94.2% (131/139), and the kappa coefficient was 0.901 (*p* < 0.001), indicating very good agreement between these two modalities (Table 1).

**Table 1:** Cross-tabulation of the diagnosis of stent compression by gray-scale imaging versus MDCTV

Gray-scale imaging	MDCTV			Sum
	No compression	Insignificant	Significant	
No compression	38	1	0	39
Insignificant	2	74	3	79
Significant	0	2	19	21
Sum	40	77	22	139

For the detection of ISR, the kappa coefficients between DUS and CEUS and between DUS and MDCTV were 0.449 and 0.516, respectively, indicating moderate agreement, while the kappa coefficient between CEUS and MDCTV was 0.884, indicating very good agreement (Tables 2, 3).

**Table 2:** Cross-tabulation of the diagnosis of ISR by DUS versus MDCTV

DUS	MDCTV		Sum
	Any degree of ISR	No ISR	
Any degree of ISR	41	9	50
No ISR	24	65	89
Sum	65	74	139

**Table 3:** Cross-tabulation of the diagnosis of ISR by CEUS versus MDCTV

CEUS	MDCTV		Sum
	Any degree of ISR	No ISR	
Any degree of ISR	59	2	61
No ISR	6	72	78
Sum	65	74	139

### Diagnostic accuracy for the detection of ISR

**Fig. 2:**
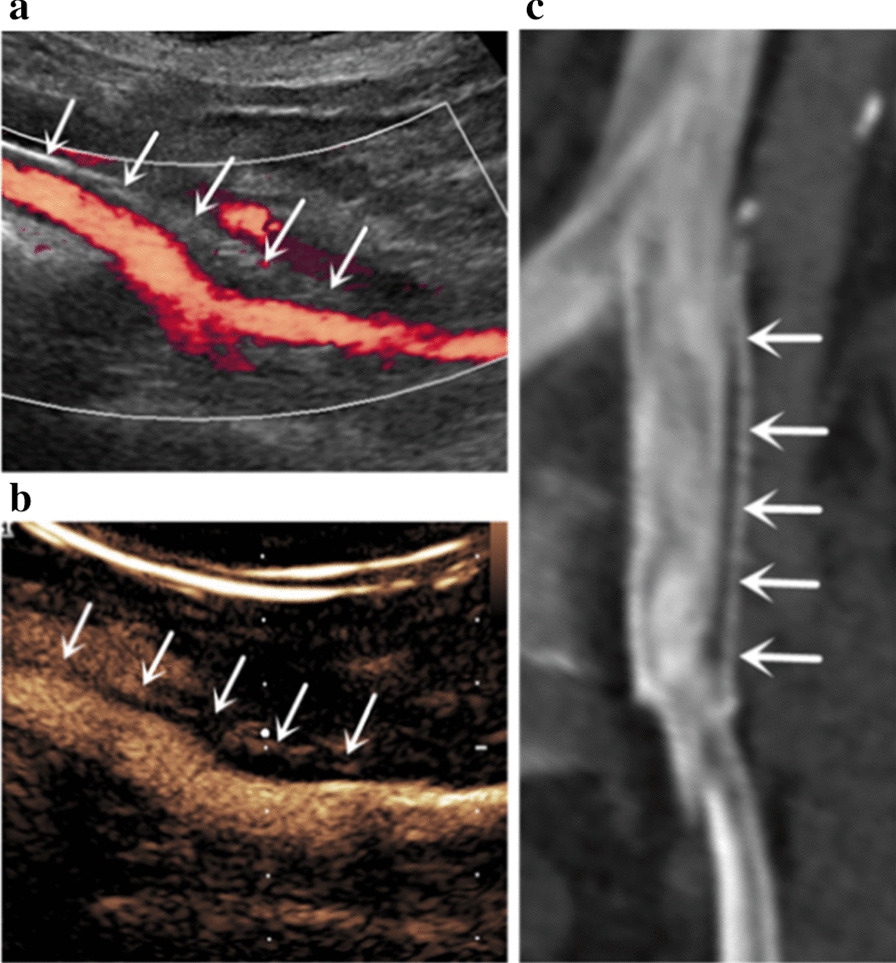
Stent ISR detected by DUS (**a**), CEUS (**b**), and MDCTV (**c**) in the follow-up of a 52-year-old male patient. In this patient, DUS (**a**) image demonstrated the presence of stent ISR (arrows), CEUS (**b**) image and MDCTV (**c**) image confirmed the diagnosis of stent ISR (arrows)

**Fig. 3:**
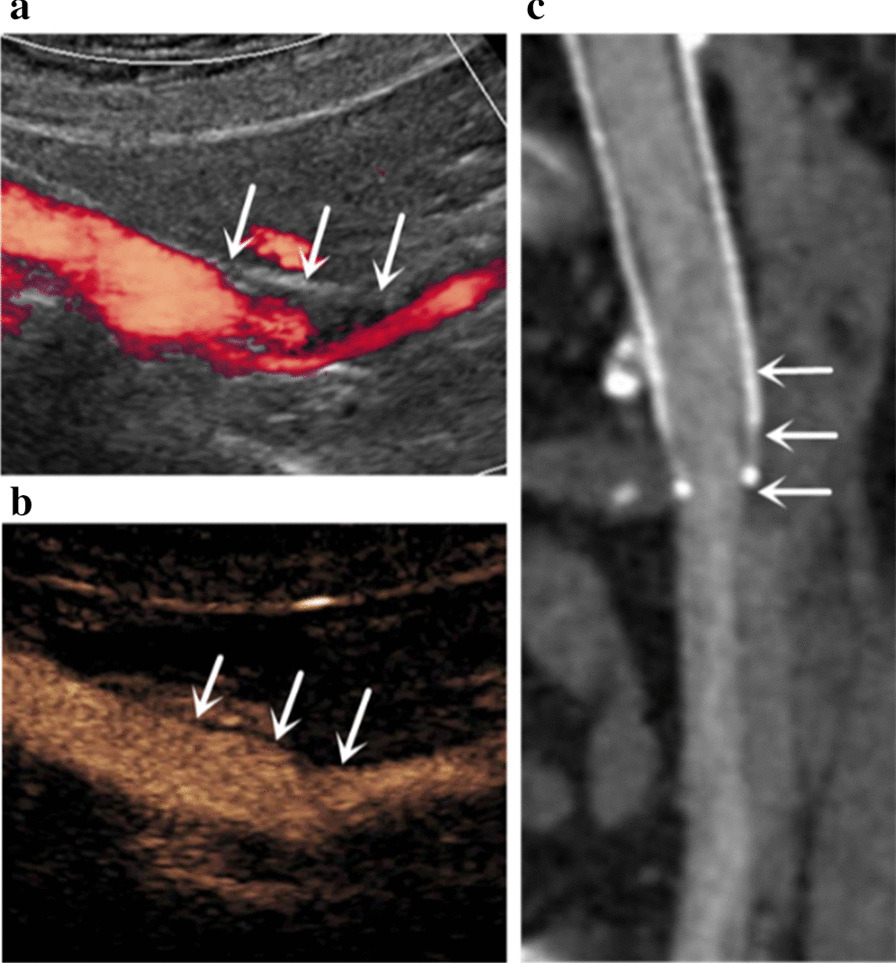
Stent ISR detected by DUS (**a**), but not by CEUS (**b**) nor MDCTV (**c**) in the follow-up of a 67-year-old female patient. In this patient, DUS (**a**) image demonstrated the presence of stent ISR (arrows), CEUS (**b**) image and MDCTV (**c**) image showed no stent ISR (arrows)

Based on the cross-tabulations of Tables 2 and 3, the sensitivity and specificity of DUS and CEUS were calculated respectively. The diagnostic accuracy values can be found in Table 4. Figure 2, 3, and 4 show the characteristic cases of stent ISR detected with DUS, CEUS, and MDCTV.

**Table 4:** Diagnostic accuracy of DUS and CEUS for the diagnosis of ISR

Parameter	DUS (%)	CEUS (%)
Sensitivity	63.1	90.8
Specificity	87.8	97.3

*DUS* Doppler ultrasound, *CEUS* contrast-enhanced ultrasound

**Fig. 4:**
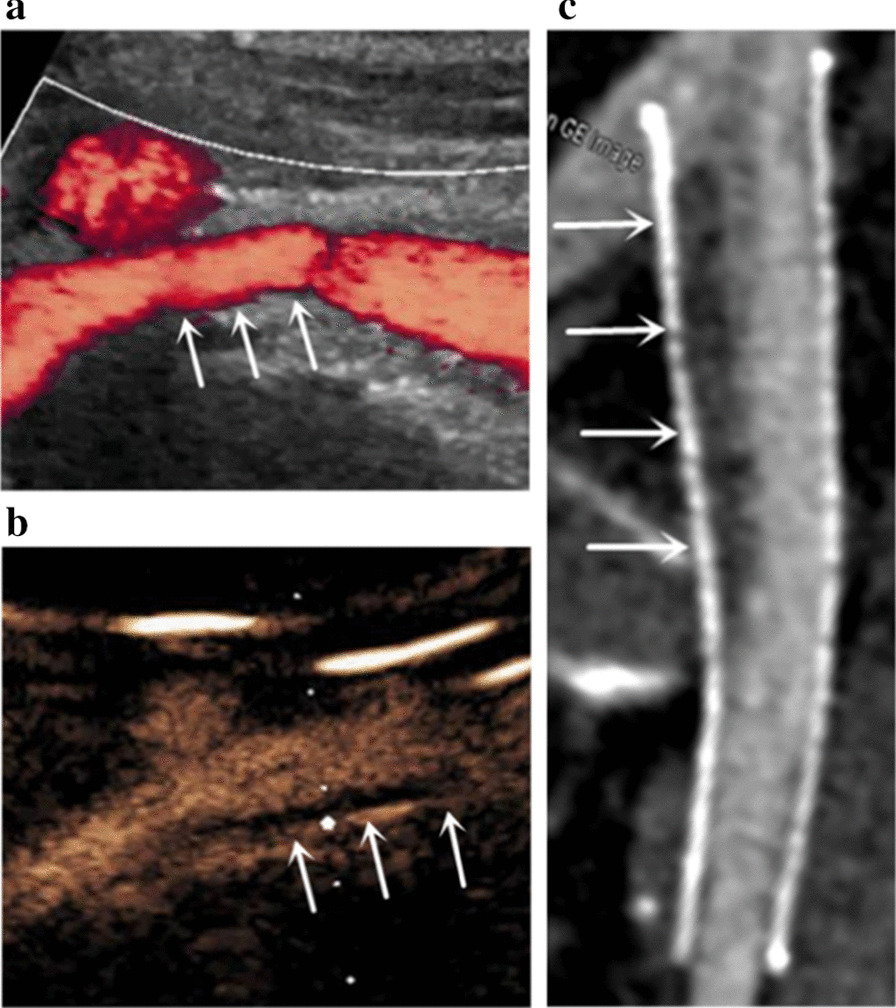
Stent ISR detected by CEUS (**b**) and MDCTV (**c**), but not by DUS (**a**) in the follow-up of a 45-year-old female patient. In this patient, DUS (**a**) image did not diagnose stent ISR (arrows), while CEUS (**b**) image and MDCTV (**c**) image revealed the presence of stent ISR (arrows)

## Discussion

The main findings of our study were that stent compression and ISR are common complications after iliac vein stenting, with estimated prevalences of 71.2% and 46.8% based on MDCTV, respectively. Ultrasound of gray-scale imaging has very good agreement with MDCTV in diagnosing stent compression. CEUS outperformed DUS in terms of diagnostic accuracy for detecting ISR.

Since there is no dedicated iliac venous stent commercially available in China, arterial-designed nitinol stents have been used as a substitute for the treatment of iliac vein obstruction. However, the arterial design includes some properties that may not suitable for the venous system [24]. A stent with sufficient radial resistive force, crush resistance and outward radial force is needed to resist the sustained compressive forces in venous compressive diseases to maintain blood flow in fibrous venous occlusions. These forces in the arterial-designed stents are most often insufficient.

Raju et al. [16] reported a rate of 25% for stent compression in 103 limbs with residual or recurrent symptoms, indicating that stent compression is a unique feature of iliac vein stenosis. An approximately oval shape can be seen at these levels of high compression, but a residual stenosis of more than 50% would be unacceptable [24]. A study [17] found that the proportion of significant stent compression was 33%; 56% of patients in the significant stent compression group developed stent occlusion, while only 9% developed stent occlusion in the insignificant group, indicating that significant compression of the nitinol stents obviously affects stent patency. Additionally, the diameter reduction caused by stent compression may result in venous hypertension and residual symptoms of lower extremity, although patency may be retained [18]. For these reasons, some researchers have pointed out that laser-cut nitinol stents might not be suitable for treating severe IVCS [20]. Our study showed that the overall incidence of laser-cut nitinol stent compression was 71.2% (99/139), with a 15.8% (22/139) incidence of significant compression based on MDCTV. The ultrasound modality of gray-scale imaging had very good agreement with MDCTV in diagnosing stent compression, which reflects that gray-scale imaging is a good alternative to MDCTV in detecting stent compression.

Another common anomalous feature of iliac venous stents is ISR. Neglén et al. [25] reported that ISR developed to some degree in 77% of limbs at 42 months, and severe (greater than 50%) stenosis was observed in 15% of limbs. A study by Raju et al. [16] showed that ISR was present in all 103 limbs with residual or recurrent symptoms. Our study also detected a high prevalence of 46.8% based on MDCTV. If a significant ISR (greater than 50%) is identified on follow-up imaging, percutaneous transluminal angioplasty (PTA) is recommended to maintain patency, especially in symptomatic patients [26].

Presently, there is no standardized noninvasive screening imaging for iliac vein stent follow-up. DUS has been the most widely used noninvasive method [27]. However, established ultrasound surveillance criteria are rarely mentioned, and independent assessments of the image quality are not provided in most studies. DUS may be insufficient because of ultrasonic attenuation by the deep location of the stents, and the orientation of the flow is almost perpendicular to the transducer. Experience indicates that DUS may be unrevealing in some cases of stent malfunction. A study [28] reported that adequate imaging of the stents could not be achieved with DUS in 23.7% of the cases. In the present study, DUS had only moderate agreement with MDCTV in detecting ISR, and the sensitivity and specificity were relatively low, only 63.1% and 87.8%, respectively. This indicates that some cases of ISR would be missed or be misdiagnosed by DUS.

With the use of contrast agents, CEUS overcomes angular dependence and the shortcoming of DUS by increasing the signal-to-noise ratio [23]. Blood flow is easy to depict with contrast material, and the visualization of the ISR is more intuitive by contrast agent filling defects. In this study, the sensitivity and specificity of CEUS were very high (over 90%), which are significantly superior to those of DUS. Thus, CEUS is an alternative modality to improve the diagnostic accuracy of DUS for the detection of ISR.

CEUS has showed real clinical value because of its advantages, such as its minimal invasiveness, rapid nature, cost-effectiveness, and good tolerability; CEUS also permits multiple follow-up surveillances without the risk of side effects. On the other hand, CEUS is characterized by some limitations that also apply to the unenhanced conventional technique. For example, attenuation because of the deep position of the stent, and obesity and bowel gas can affect the imaging [23]. However, in our experience, as long as adequate intestinal preparation is accomplished, CEUS is easy to perform, and good image quality can be obtained. Furthermore, CEUS is operator-dependent, standardized training and specific skills are required to obtain quality images.

Our study has some limitations. First, as the reasons mentioned earlier, we could not use IVUS as the gold standard, which may affect the real diagnostic accuracy of non-invasive technique of CEUS. In clinical practice, only a few patients with indications of re-intervention were willing to receive further treatment, leading the small number of cases that could be confirmed by IVUS.

Second, the absence of hemodynamic parameters like peak velocity or velocity index or velocity ratio may be a weakness of the study [29]. However, visualization of the blood flow within the stent is necessary to assess stent patency, the abnormalities of hemodynamic parameters detected by pulsed-wave Doppler are useful but are of limited value for the quantification of lesion severity [30].

Third, in this study, all ultrasonographic examinations were performed by a single ultrasonologist. Therefore, the reproducibility was unknown.

The last possible drawback was that the specific degree of ISR and the overall degree of stenosis when stent compression presents with ISR was not assessed. Measuring diameter stenosis by DUS and MDCTV is problematic because of the irregular shape of neoplasms and the elliptical shape of the post-stented lumen. The optimal assessment would be to use IVUS to directly delineate the area encompassed by the stent perimeter and then calculate the percentage stenosis with the lumen area within the ISR [16].

## Conclusion

CEUS can directly visualize the blood flow throughout the entire stent, which constitutes a valuable complementary technique with superior diagnostic accuracy for the follow-up of iliac vein stent.

## Data Availability

The data that support the findings of this study are available from the corresponding author on reasonable request.

## Abbreviations

DUS: Doppler ultrasound
CEUS: Contrast-enhanced ultrasound
MDCTV: Multidetector computed tomography venography
ISR: In-stent restenosis
IVUS: Intravascular ultrasound
IVCS: Iliac vein compression syndrome
PTS: Post-thrombotic syndrome
IVC: Inferior vena cava
DVT: Deep venous thrombosis
PTA: Percutaneous transluminal angioplasty

## References

[1] Shi WY, Gu JP, Liu CJ, He X, Lou WS (2016). Endovascular treatment for iliac vein compression syndrome with or without lower extremity deep vein thrombosis: a retrospective study on mid-term in-stent patency from a single center. Eur J Radiol.

[2] Xue GH, Huang XZ, Ye M, Liang W, Zhang H, Zhang JW (2014). Catheter-directed thrombolysis and stenting in the treatment of iliac vein compression syndrome with acute iliofemoral deep vein thrombosis: outcome and follow-up. Ann Vasc Surg.

[3] Jayaraj A, Crim W, Knight A, Raju S (2019). Characteristics and outcomes of stent occlusion after iliocaval stenting. J Vasc Surg Venous Lymphat Disord.

[4] Rollo JC, Farley SM, Oskowitz AZ, Woo K, DeRubertis BG (2017). Contemporary outcomes after venography-guided treatment of patients with May–Thurner syndrome. J Vasc Surg Venous Lymphat Disord.

[5] van Vuuren TMAJ, Doganci S, Wittens CHA (2018). Patency rates and clinical outcomes in a cohort of 200 patients treated with a dedicated venous stent. J Vasc Surg Venous Lymphat Disord.

[6] Rossi FH, Kambara AM, Izukawa NM, Rodrigues TO, Rossi CB, Sousa AG (2018). Randomized double-blinded study comparing medical treatment versus iliac vein stenting in chronic venous disease. J Vasc Surg Venous Lymphat Disord.

[7] Pokrovskiĭ AV, Ignat'ev IM, Gradusov EG (2018). Results of endovascular treatment of obstructive lesions of veins of the iliofemoral segment. Angiol Sosud Khir.

[8] Abdul-Haqq R, Novak Z, Pearce BJ, Matthews TC, Patterson MA, Jordan WD (2017). Routine extended follow-up surveillance of iliac vein stents for iliocaval venous obstruction may not be warranted. J Vasc Surg Venous Lymphat Disord.

[9] Black S, Gwozdz A, Karunanithy N, Silickas J, Breen K, Hunt B (2018). Two year outcome after chronic iliac vein occlusion recanalisation using the Vici venous stent. Eur J Vasc Endovas Surg.

[10] Razavi M, Marston W, Black S, Bentley D, Neglén P (2018). The initial report on 1-year outcomes of the feasibility study of the Veniti Vici Venous Stent in symptomatic iliofemoral venous obstruction. J Vasc Surg Venous Lymphat Disord.

[11] Stuck AK, Reich T, Engelberger RP, Sebastian T, Kucher N (2018). Endovascular treatment of post-thrombotic and non-thrombotic iliofemoral venous outflow obstructions with self-expanding nitinol stents. Vasa.

[12] Lichtenberg M, de Graaf R, Stahlhoff WF, Özkapi A, Simon M, Breuckmann F (2019). Patency rates, safety and clinical results of the sinus-Obliquus venous stent in the treatment of chronic ilio-femoral venous outflow obstruction-data from the Arnsberg venous registry. Vasa.

[13] Seager MJ, Busuttil A, Dharmarajah B, Davies AH (2016). Editor's choice—a systematic review of endovenous stenting in chronic venous disease secondary to iliac vein obstruction. Eur J Vasc Endovasc Surg.

[14] Razavi MK, Jaff MR, Miller LE (2015). Safety and effectiveness of stent placement for iliofemoral venous outflow obstruction: systematic review and meta-analysis. Circ Cardiovasc Interv.

[15] Wen-da W, Yu Z, Yue-Xin C (2016). Stenting for chronic obstructive venous disease: a current comprehensive meta-analysis and systematic review. Phlebology.

[16] Raju S, Davis M (2014). Anomalous features of iliac vein stenosis that affect diagnosis and treatment. J Vasc Surg Venous Lymphat Disord.

[17] Cho H, Kim JW, Hong YS, Lim SH, Won JH (2015). Stent compression in iliac vein compression syndrome associated with acute ilio-femoral deep vein thrombosis. Korean J Radiol.

[18] Raju S, Tackett PJ, Neglen P (2009). Reinterventions for nonocclusive iliofemoral venous stent malfunctions. J Vasc Surg.

[19] Gagne PJ, Gasparis A, Black S, Thorpe P, Passman M, Vedantham S (2018). Analysis of threshold stenosis by multiplanar venogram and intravascular ultrasound examination for predicting clinical improvement after iliofemoral vein stenting in the VIDIO trial. J Vasc Surg Venous Lymphat Disord.

[20] Jeon UB, Chung JW, Jae HJ, Kim HC, Kim SJ, Ha J (2010). May–Thurner syndrome complicated by acute iliofemoral vein thrombosis: helical CT venography for evaluation of long-term stent patency and changes in the iliac vein. AJR Am J Roentgenol.

[21] Metzger PB, Rossi FH, Kambara AM (2016). Criteria for detecting significant chronic iliac venous obstructions with duplex ultrasound. J Vasc Surg Venous Lymphat Disord.

[22] Bredahl KK, Taudorf M, Lönn L, Vogt KC, Sillesen H, Eiberg JP (2016). Contrast enhanced ultrasound can replace computed tomography angiography for surveillance after endovascular aortic aneurysm repair. Eur J Vasc Endovasc Surg.

[23] Micol C, Marsot J, Boublay N, Pilleul F, Berthezene Y, Rode A (2012). Contrast-enhanced ultrasound: a new method for TIPS follow-up. Abdom Imaging.

[24] de Wolf MA, de Graaf R, Kurstjens RL, Penninx S, Jalaie H, Wittens CH (2015). Short-term clinical experience with a dedicated venous nitinol stent: initial results with the sinus-venous stent. Eur J Vasc Endovasc Surg.

[25] Neglén P, Raju S (2004). In-stent recurrent stenosis in stents placed in the lower extremity venous outflow tract. J Vasc Surg.

[26] Abou Ali AN, Avgerinos ED, Chaer RA (2018). Role of venous stenting for iliofemoral and vena cava venous obstruction. Surg Clin North Am.

[27] Hage AN, Srinivasa RN, Abramowitz SD, Gemmete JJ, Reddy SN, Chick JFB (2018). Endovascular iliocaval stent reconstruction for iliocaval thrombosis: a multi-institutional international practice pattern survey. Ann Vasc Surg.

[28] Ye K, Lu X, Jiang M, Yang X, Li W, Huang Y (2014). Technical details and clinical outcomes of transpopliteal venous stent placement for postthrombotic chronic total occlusion of the iliofemoral vein. J Vasc Interv Radiol.

[29] Sebastian T, Barco S, Engelberger RP, Spirk D, Schindewolf M, Baumann F (2020). Duplex ultrasound investigation for the detection of obstructed iliocaval venous stents. Eur J Vasc Endovasc Surg.

[30] Sloves J, Almeida JI (2018). Venous duplex ultrasound protocol for iliocaval disease. J Vasc Surg Venous Lymphat Disord.

